# Cannabinoid 1 receptor signaling on GABAergic neurons influences astrocytes in the ageing brain

**DOI:** 10.1371/journal.pone.0202566

**Published:** 2018-08-16

**Authors:** Andras Bilkei-Gorzo, Onder Albayram, Frank Ativie, Safak Chasan, Till Zimmer, Karsten Bach, Andreas Zimmer

**Affiliations:** Institute of Molecular Psychiatry, Medical Faculty of the University of Bonn, Bonn, Germany; University of Modena and Reggio Emilia, ITALY

## Abstract

Astrocytes, key regulators of brain homeostasis, interact with neighboring glial cells, neurons and the vasculature through complex processes involving different signaling pathways. It is not entirely clear how these interactions change in the ageing brain and which factors influence astrocyte ageing. Here, we investigate the role of endocannabinoid signaling, because it is an important modulator of neuron and astrocyte functions, as well as brain ageing. We demonstrate that mice with a specific deletion of CB1 receptors on GABAergic neurons (GABA-Cnr1^-/-^ mice), which show a phenotype of accelerated brain ageing, affects age-related changes in the morphology of astrocytes in the hippocampus. Thus, GABA-Cnr1^-/-^ mice showed a much more pronounced age-related and layer-specific increase in GFAP-positive areas in the hippocampus compared to wild-type animals. The number of astrocytes, in contrast, was similar between the two genotypes. Astrocytes in the hippocampus of old GABA-Cnr1^-/-^ mice also showed a different morphology with enhanced GFAP-positive process branching and a less polarized intrahippocampal distribution. Furthermore, astrocytic TNFα levels were higher in GABA-Cnr1^-/-^ mice, indicating that these morphological changes were accompanied by a more pro-inflammatory function. These findings demonstrate that the disruption of endocannabinoid signaling on GABAergic neurons is accompanied by functional changes in astrocyte activity, which are relevant to brain ageing.

## Introduction

Around 60% of the axon-dendritic synapses in the hippocampus are surrounded by astrocytic processes [1]. This enables astrocytes to support synaptogenesis [2] and synaptic activity through the synthesis, uptake, and recycling of glutamate [3] as well as the release of astrocytic transmitters like glutamate [4], D-serine [5], GABA [6], and purines [7]. Astrocytes also influence synaptic activity indirectly by providing energy support for neurons [8], regulating ion homeostasis [9], and neuronal excitability [10]. Thus, changes in astrocyte activity during ageing may influence brain functions. Indeed, the ageing brain exhibits characteristic changes in synaptic plasticity and metabolic balance, which are known to be regulated by astrocytes [11].

During normal, healthy ageing astrocytes undergo characteristic morphological and functional changes, characterized by an elevated expression of inflammatory cytokines [12] and glial fibrillary acidic protein (GFAP) [13].

It has been hypothesized that the age-related increase in GFAP expression is associated with reduced neuroprotective capacity [11]. During ageing, an increasing number of astrocytes switch from a resting-quiescent state to a mild-to-moderate activated or hypertrophic state, which renders astrocytes to relinquish some of their neurosupportive activities [14]. A recent study focusing on the transcriptome of astrocytes revealed that genes involved in synapse elimination and immune response are upregulated during ageing [15]. All these changes are similar to those observed during inflammatory reactions [16]. The activation of astrocytes during pathological conditions has been extensively studied [17], but it is not fully known which factors influence the development of similar changes during healthy ageing.

Several lines of evidence suggest that the cannabinoid system influences the ageing process in the brain and other organs. Young mice with a deletion of CB1 receptors (Cnr1^-/-^) show a superior performance in behavioral models of learning and memory [18,19] and enhanced long-term potentiation [20]. However, 6-month-old knockouts already display cognitive deficits [21], which become very severe at the age of 12-months [18,22]. The learning deficits in the 12-month-old CB1^-/-^ mice were accompanied by neuroinflammatory changes. Interestingly, genetic deletion of CB1 receptors from GABAergic neurons led to similar inflammatory changes [22] suggesting that GABAergic neurons have a key role in the regulation of glial activity. Recent evidence also suggests that endocannabinoid signaling is involved in the bidirectional communication between neurons and glia cells [23–25]. Astroglial cells produce the major ligand of CB1 receptors, 2-arachidonoylglycerol (2-AG) [26], whereas GABAergic neurons express CB1 receptors on the highest level in the hippocampus [27,28].

Mice with selective deletion of CB1 receptors on GABAergic neurons (here called GABA-Cnr1^−/−^) appear on first sight healthy and breed well. Electrophysiological studies however revealed that depolarization-induced depression of inhibition (DSI) was totally abolished [29] and long term potentiation (LTP) decreased [30] in the hippocampus of conditional mutants. As a consequence, these animals show deficits in hippocampal learning [31] and stress coping [32]. GABA-Cnr1^−/−^ animals have similar body weight, food intake [33] and stress reactivity [34] as wild type controls. The behavioral phenotype of this mouse strain was rather mild and included a decreased wheel-running performance [35], enhanced sensitivity to cocaine [36] and in males increased social preference to females [37]. Histological studies also found no difference in the density of GABAergic neurons, nor alterations in specific GABAergic neuron subtypes [31]. However, aged GABA-Cnr1^−/−^ mice showed an increase in GFAP-positive astrocyte-covered areas in the hippocampus, a higher density of activated microglia, and an enhanced expression of the inflammatory cytokines TNFα and IL-6 when compared to wild-type littermates [22].

In the present work we asked whether CB1 signaling on GABAergic neurons affects the dynamics of age-related changes in astrocytes. To answer this question we compared astrocyte morphology, distribution and production of the pro-inflammatory cytokine TNFα in the hippocampus between 4 age groups of wild-type and GABA-Cnr1^-/-^ mice.

## Materials and methods

### Animals

Male and female mice aged 2, 6, 12 and 24 months were used in all experiments. Animals were housed under a reversed light cycle in groups of 3–5 with food and water ad libitum. GABA-Cnr1^−/−^ mice (B6.cg Cnr1 tm1.2Ltz x Tg(dlx6a-cre)1Mekk; [29] and wild type litter-mates on a C57BL/6N genetic background were kindly provided by Beat Lutz (University of Mainz, Germany). Care of the animals and execution of all experiments followed the guidelines of European Communities Directive 2010/63/EU and the German Animal Protection Law regulating animal research. The protocol was approved by the local Committee on the Ethics of Animal Experiments of Landesamt für Natur, Umwelt und Verbraucherschutz Nordrhein-Westfalen (LANUV NRW; Permission Number: 84–02.04.2015.A192). The animals were deeply anesthetized before fixation with isoflurane, and all efforts were made to minimize suffering.

### Tissue preparation

Animals were deeply anaesthetized with isoflurane and transcardially perfused with 4% formaldehyde solution. Brains from these animals were isolated, kept in 30% sucrose solution for 24 hours for cryoprotection, snap frozen in dry ice-cooled isopentane, and stored in -80°C until further processing. Next, coronal slices of the hippocampal-regions were serially sectioned at 18-μm using a microtome at -20°C (Leica CM 3050; Leica Microsystems) and mounted onto silanized glass slides. Glass slides were kept at −80°C until further use.

### Immunofluorescence staining

Frozen sections were immediately dried after removal from the freezer for 20–30 min at 40°C on a hot plate. After drying, the slices were framed with a PapPen, washed in PBS for 5 min at room temperature and permeabilised in PBS containing 0.5% Triton X-100 for 30 min at room temperature. After two more five-minute washes in PBS, nonspecific binding was blocked by incubation in PBS containing 5% normal donkey serum (NDS) or 5% normal goat serum (NGS) and 3% bovine serum albumin (BSA) for 1 h. For the GFAP/S100β co-staining (experimental group 1), the slices were incubated with rabbit anti-S100β antibody solution (Abcam; 1:2000 diluted in 3% BSA containing PBS and with a mouse anti-GFAP antibody solution (Abcam; 1:2000 diluted in 3% BSA/PBS) for 48 hours. For the GFAP/TNFα co-staining (experimental group 2), rabbit anti-GFAP (Abcam; 1:1000) and mouse anti-TNFα antibody solution (Abcam; 1:100 diluted in 3% BSA/PBS) were used with a 24 h incubation time at 4°C. Afterwards, slides were washed three times for 10 min in PBS at room temperature, followed by incubation with the respective secondary antibody for 1 h at room temperature. These were AF 488-conjugated donkey anti-mouse IgG (Invitrogen; 1:500 diluted in 3% BSA/PBS); Cy3-conjugated goat anti-rabbit (Jackson; 1:500 diluted in 3% BSA/PBS); AF 488-conjugated donkey anti-rabbit IgG (Invitrogen; 1:2000 diluted in 3% BSA/PBS) and AF 647-conjugated donkey anti-mouse IgG (Invitrogen; 1:1000 diluted in 3% BSA/PBS). Then, slides were washed three times for 10 min in PBS and in milli-Q water for 1 min. After staining, the slices were mounted with 4',6-diamidino-2-phenylindole (DAPI) and covered. Fluorescence images were obtained with a Zeiss Axiovert 200 M fluorescent microscope (Carl Zeiss Microimageing) with 20x objective lens. For testing TNFα within GFAP-positive astrocytes we used a Leica LSM SP8 confocal microscope with a 63x lens.

### Determination of astrocyte densities as GFAP-positive areas

Brains of the animals were sliced, stained and analysed in two separate sets of experiments by two experienced investigators. In both experimental groups, 2–3 animals/group were analysed so the animal number is 5–6 per age and genotype. The area covered by GFAP-immunoreactive astrocytes was determined by the area fraction technique [38] using the ImageJ software (1.43r) in the first and with Fiji 1.0 in the second experimental group. In both cases, the percentage of GFAP-positive areas (signal intensity above a threshold, which was the same for all the probes within the experimental group) was calculated in the CA1 and CA3 regions of the hippocampus. To analyse the age- and genotype-related changes in the distribution of astrocytes, the percentage of GFAP-positive areas was determined in a layer-specific manner in the stratum oriens (so), pyramidale (sp), stratum lucidum (sl), stratum radiate (sr) and stratum lacunosum-moleculare (slm) layers of the CA3 region and in the so, sp, sr and slm layers in the CA1 region [39,40]. For the identification of GFAP-positive areas the same background value was used for all the slices within the same staining session; the investigator was blinded to the group. To note: old animals had higher lipofuscin level, which gave a characteristic intrinsic fluorescent signal, therefore the microscopic picture of old and young animals clearly differed. GFAP-positive areas of the regions of interests were determined on six sections per animal, mean values were calculated and used for further analysis.

### Stereological quantification of S100β-positive astrocytes

For stereological quantification, every 10th slice of the region of interest was selected and stained for S100β immunoreactivity. In this way, we analysed 10–15 sections per region of interest. Microscopy was performed with a motorized x-y-z stage coupled to a Zeiss Axiovert 200M fluorescent microscope equipped with a Zeiss ApoTome (Carl Zeiss Microimageing, Oberkochen, Germany) over the hippocampus [41]. The total number of S100β-immunoreactive astrocytes in the CA1 and CA3 regions of the hippocampus was estimated by using the optical fractionator technique as described previously [42,43]. To assess changes in the distribution of astrocytes, we determined the number of S100β-positive cell bodies on a layer-specific manner in the CA1 and CA3 regions.

### Quantitative morphological analyses of GFAP-positive astrocytic cell processes

The morphology of GFAP- and S100β-immunopositive astrocytes was assessed with a 63x, 1.4 NA oil-immersion lens. Astroglial cells were identified as S100β-immunoreactive cells with rod-shape cell bodies (2–6 μm in diameter) and ramified proximal processes with GFAP immunoreactivity [44]. Counting of the branching of all GFAP-positive main processes up to 15 μm from the center of each astrocytic soma was performed using the ImageJ software in 388 randomly selected cells [45,46]. Background staining intensities were identical in all samples, therefore we used the same threshold value for the determination of GFAP-positive processes.

### Analyses of astrocytic TNFα expression

Samples were stained and analysed in two separate experiments by two different experienced investigators. The co-expression of GFAP- and TNFα was assessed with a 63x water-immersion lens using Leica LSM SP8 confocal microscope. The intensity of TNFα immunoreactivity was determined in astrocytes (50–200 μm^2^ GFAP-positive areas, which are directly adjacent or containing DAPI-positive nuclei) using the Fiji software by two separate investigators, who were blinded to age or genotype.

### Real-time PCR analysis of the hippocampal TNFα expression

A separate group of 2-, 6-, 12- and 24-month old animals (10–18 per age-group and genotype) was used for this study. The animals were decapitated, their brains removed, hippocampi isolated and snap-frozen in isopentane. The samples were lysed in TRIzol (Life Technologies), and total RNA was extracted according to the manufacturer’s protocol. The quality of the RNA was assessed by measuring the ratio of the absorbance at 260 nm and 280 nm using a Nanodrop 2000 Spectrometer (Thermo Scientific). Probes with a 260/280 ratio less than 1.9 were rejected. cDNAs were synthesized using the SuperScript First-Strand Synthesis System for RT-PCR Kit (Invitrogen Corp., Carlsbad, CA, USA) with random hexamer primers. Total RNA (0.6 μg) was used as starting material for cDNA synthesis. Differences in mRNA expression were determined in triplicate by custom TaqMan® Gene Expression Assays (Applied Biosystems, Darmstadt, Germany; tumor necrosis factor (TNF): Mm00443258_m1. The 3-phosphate dehydrogenase (GAPDH): Mm01334042_m1 was used as an endogenous reference gene to standardize the amount of target cDNA. Typically, a reaction mixture consisted of 1x TaqMan® Gene Expression Master Mix (Applied Biosystems, Darmstadt, Germany), 2 μl cDNA and 1x Custom TaqMan® Gene Expression Assay. Samples were processed in a 7500 Real-Time PCR Detection System (Applied Biosystems, Darmstadt, Germany) with the following cycling parameters: 95°C for 10 min (hot start), 40 cycles at 95°C for 15 s (melting) and 60°C for one minute (annealing and extension). Analysis was performed using the 7500 Sequence Detection Software version 2.2.2 (Applied Biosystems, Darmstadt, Germany) and data were obtained as function of threshold cycle (Ct). Relative quantitative gene expression was calculated with the 2^-dCt^ method. Briefly: dCt was calculated for each assayed sample by subtracting Ct of the housekeeping gene from the Ct of TNFα.

### Statistics

Distribution of the data was analyzed using D´Agostino & Pearson normality test, when the case number was 20 or more in the individual groups. Two-way ANOVA followed by Bonferroni test was used to compare astrocyte numbers, number of astrocyte processes, TNFα immunoreactivity and expression between genotypes and age-groups. Results of the two-way ANOVA analyses are presented as bar diagrams, where columns represent mean values, error bars standard error of means (SEM). Distribution of astrocytes was analysed using three-way ANOVA with genotype, age and layer as main factors, the individual groups were compared using Bonferroni´s t-test. Results of the three-way ANOVA analyses are presented as line diagrams, where symbols indicate mean values and error bars SEM. To compare the size of GFAP-covered areas between age groups and genotypes, data were first analysed using three-way ANOVA (main effects: experimental group, age, and genotype). To test if hippocampal layers are similarly affected we used four-way repeated ANOVA (between effects: experimental group, age, genotype; within effect: layer). We found no interaction between experimental group and any other parameter, therefore we merged the results from the two sets of experiments and analysed using two- way (age and genotype effect) or three-way (genotype, age and layer effect) ANOVA as described above. The relation between TNFα levels and GFAP-positive process number was analysed by Pearson correlation analysis. Data for the correlation analysis are presented as dot plot. P values less than 0.05 were selected as statistically significant.

## Results

### Age-related changes in the number and distribution of astrocytes

**Fig 1:**
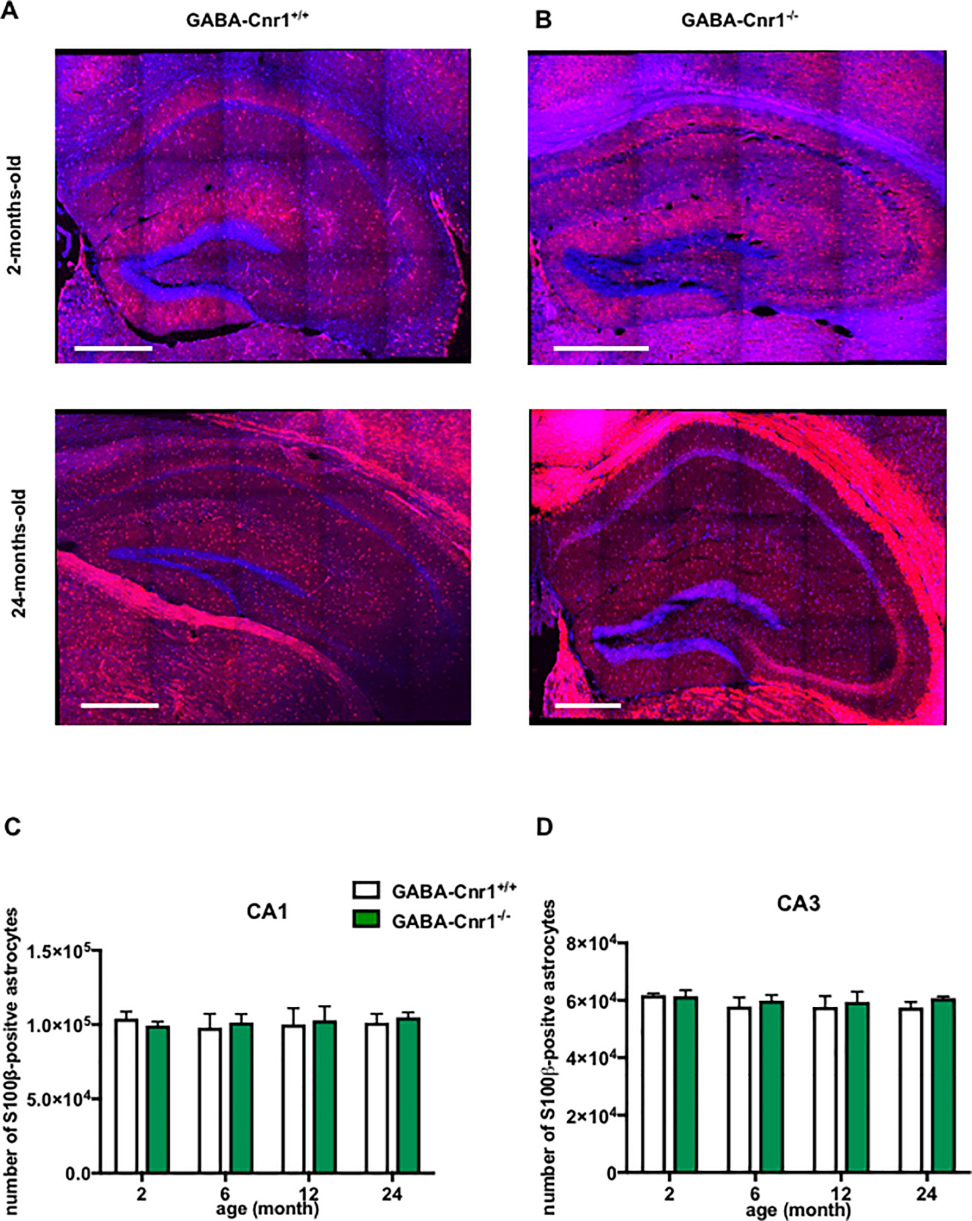
Representative photomicrographs of s100β-immunopositive astrocytic soma in the hippocampus of **(A)** GABA-Cnr1^+/+^ 2- months-old (upper panel) and 24-months-old (lower panel) and **(B)** GABA-Cnr1^−/−^ 2- months-old (upper panel) and 24-months-old (lower panel) mice. Scale bars represent 500 μm. Quantitative stereological analysis of the total number of S100β-immunopositive astrocytic soma within hippocampal **(C)** CA1 and **(D)** CA3 regions in 2-, 6-, 12- and 24-month-old GABA-Cnr1^-/-^ and GABA-Cnr1^+/+^ littermates. (n = 3 per age and genotype). Columns represent mean values, error bars standard error of means (SEM).

**Fig 2:**
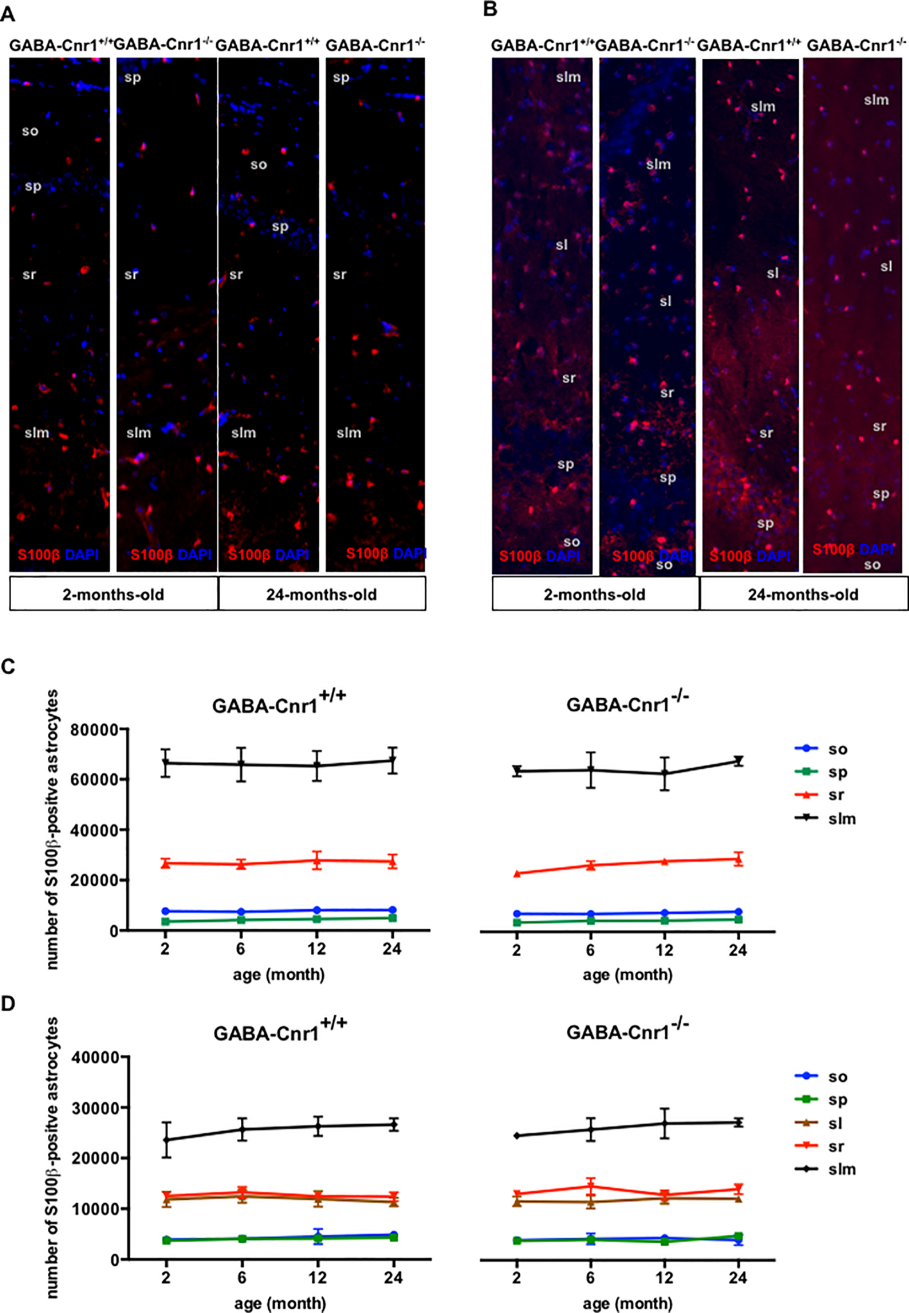
Representative photomicrographs of s100β-immunopositive astrocytic soma in the hippocampal CA1 **(A)** and CA3 **(B)** layers from 2- and 24-months-old GABA-Cnr1^+/+^ (right panel) and GABA-Cnr1^−/−^ (left panel) mice. The width of the panels is 100 μm. (so) stratum oriens, (sp) stratum pyramidale, (sl) stratum lucidum, (sr) stratum radiate, (slm) stratum lacunosum-moleculare. Spatial distribution of the total number of s100β-immunopositive astrocytic soma in the CA1 **(C)** and CA3 **(D)** regions of the hippocampus in 2-, 6-, 12- and 24-month-old GABA-Cnr1^-/-^ and GABA-Cnr1^+/+^ littermates (n = 3 per age and genotype). Symbols indicate mean values and error bars standard error of means.

To determine changes in astrocyte numbers we first utilised S100β-specific antibodies. S100β is a perinuclear protein in astrocytes, which permits the precise localization of astrocyte cell bodies [45]. First, we compared the number and spatial distribution of S100β-immunopositive astrocytic somata within the layers of the hippocampal CA1 & CA3 regions in 2-, 6-, 12- and 24-months-old GABA-Cnr1^-/-^ and GABA-Cnr1^+/+^ littermates (Fig 1A and 1B). As shown in Fig 1C and 1D, stereological analysis revealed that the number of S100β-immunopositive astrocytes was similar in all age groups (CA1 region: F _3,6_ = 0.071, p > 0.05; CA3 region: F _3,6_ = 0.8732, p > 0.05) and not affected by the genotype (CA1 region: F _1,6_ = 0.0786, p > 0.05; CA3 region: F _1,6_ = 0.6291, p > 0.05). A detailed analysis of astrocytic somata in different hippocampal cell layers showed an uneven distribution (main effect: layer—CA1 region: F _3,18_ = 695.8, p *<* 0.001; CA3 region: F _4,24_ = 340.7, p *<* 0.001), with astrocytes being most abundantly positioned in the slm and least abundantly in the pr layer (Fig 2A and 2B). The spatial pattern of distribution was neither influenced by age (interaction: layer x age—CA1 region: F _9,18_ = 0.181, p *>* 0.05; CA3 region: F _12,24_ = 0.460, p *>* 0.05) nor genotype (interaction: layer x genotype—CA1 region: F _3,16_ = 0.009, p *>* 0.05; CA3 region: F _3,25_ = 0.403, p *>* 0.05) (Fig 2C and 2D). There was also no interaction between these factors (interaction: layer x age x genotype—CA1 region: F _9,18_ = 0.077, p *>* 0.05; CA3 region: F _12,24_ = 0.067, p *>* 0.05) (Fig 2A and 2B).

**Fig 3:**
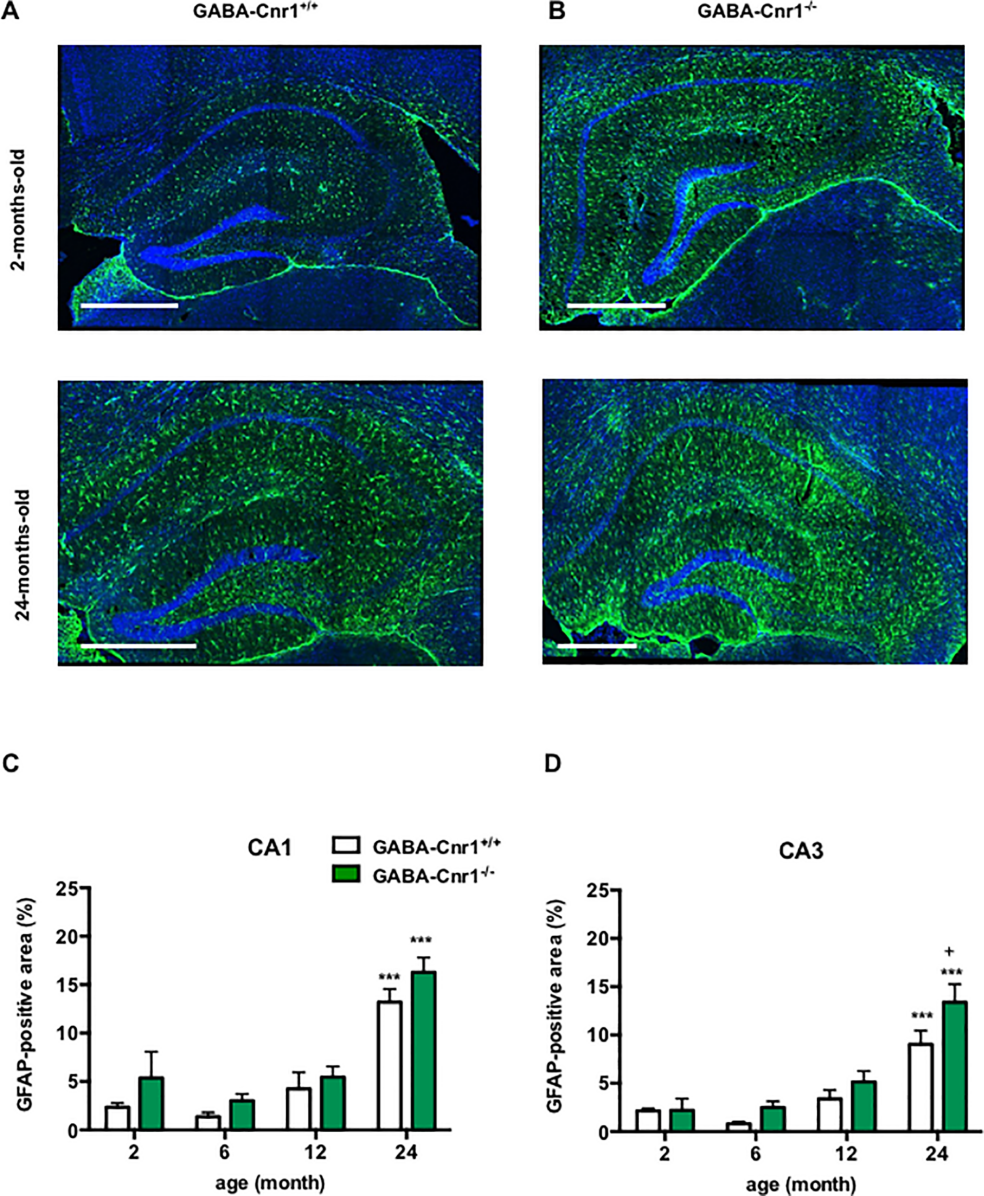
Representative photomicrographs of GFAP-immunostaining in the hippocampus of **(A)** GABA-Cnr1^+/+^ 2- months-old (upper panel) and 24-months-old (lower panel) and **(B)** GABA-Cnr1^−/−^ 2- months-old (upper panel) and 24-months-old (lower panel) mice. Scale bars represent 500 μm. Density of the GFAP-immunopositive areas within hippocampal **(C)** CA1 and **(D)** CA3 regions in 2-, 6-, 12- and 24-months-old GABA-Cnr1^+/+^ and GABA-Cnr1^-/-^ littermates. *** p < 0.001 significantly different compared to 2-months-old mice from the same genotype. + p < 0.05 significantly different compared to GABA-Cnr1^+/+^ mice from the same age group. n = 5–6 per age group and genotype Columns represent mean values, error bars standard error of means (SEM).

We next determined the size of the areas covered by GFAP-immunopositive astrocytes in the hippocampus. Increased GFAP expression in the brain of older individuals is easily detectable using immunohistological staining as enhanced GFAP-positive area [22], which is generally interpreted as a sign of enhanced astrocyte activity [47]. The two separate sets of experiments provided the same results, therefore the data were merged, analysed and presented together (Fig 3A and 3B). We found a main genotype effect for the size of GFAP-positive areas (genotype effect: CA1 region: F _1,29_ = 10.71, p < 0.01 CA3 region: F _1,28_ = 6.388, p *<* 0.05) (Fig 3C and 3D). Post hoc analysis using Bonferroni t-test showed that the size of GFAP-positive areas was higher in 24-month-old GABA-Cnr1^-/-^ animals compared to their wild-type siblings (Fig 3D). We also found an age-related increase in the size of GFAP-immunopositive areas (CA1 region: F _3,29_ = 57.54, p < 0.001; CA3 region: F _3,28_ = 35.34, p < 0.001), but no genotype x age interaction (CA1 region: F _3,29_ = 0.178, p *>* 0.05; CA3 region: F _3,28_ = 0.042, p *>* 0.05) (Fig 3C).

**Fig 4:**
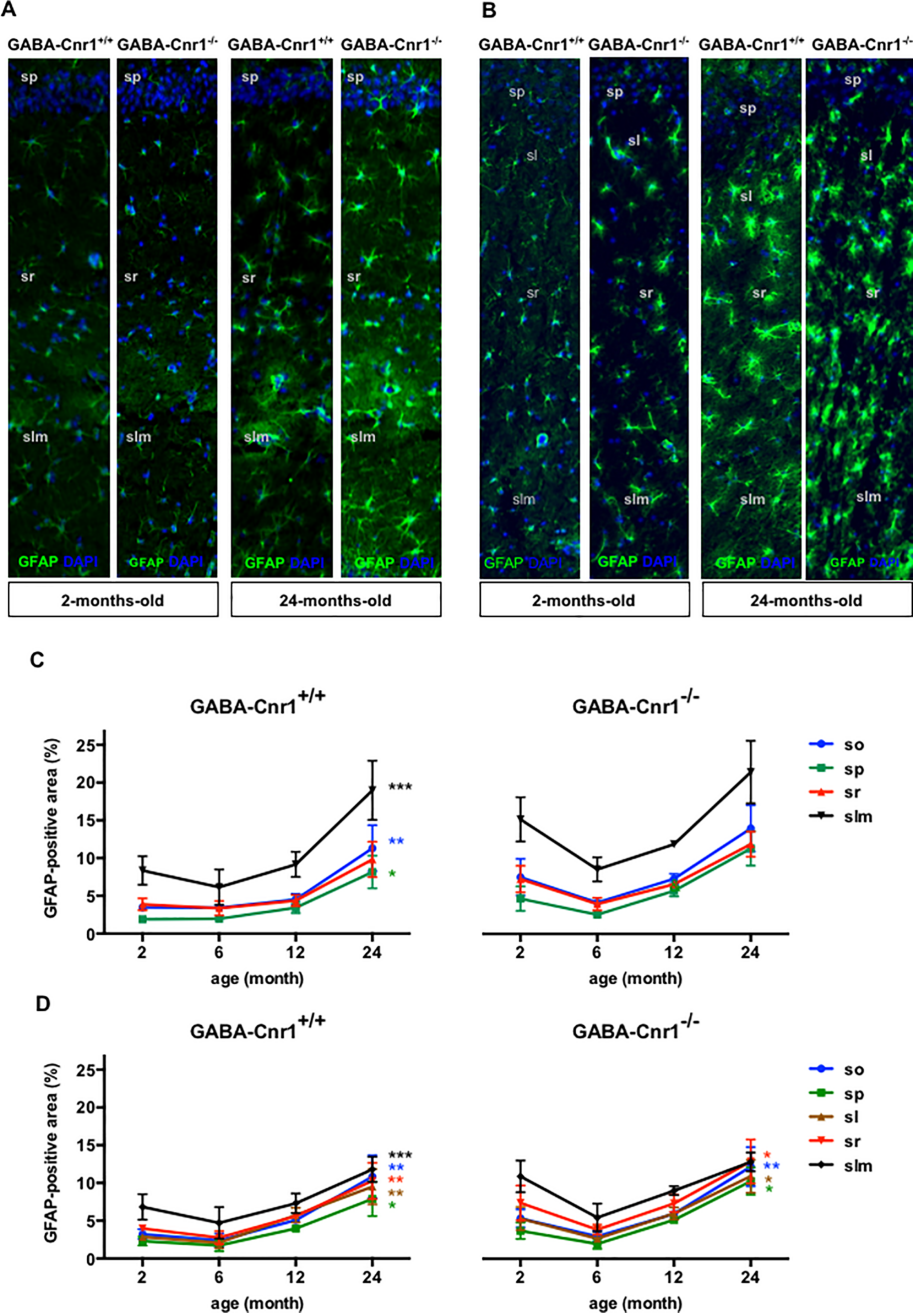
Representative photomicrographs of GFAP-immunopositive astrocytes in the hippocampal CA1 **(A)** and CA3 **(B)** layers from 2- and 24-months-old GABA-Cnr1^+/+^ and GABA-Cnr1^−/−^ mice. The width of the panels is 100 μm. (sp) stratum pyramidale, (sl) stratum lucidum, (sr) stratum radiate, (slm) stratum lacunosum-moleculare. Spatial distribution of GFAP-immunopositive areas within layers of the hippocampal CA1 **(C)** and CA3 **(D)** regions in 2-, 6-, 12- and 24-month-old GABA-Cnr1^-/-^ and GABA-Cnr1^+/+^ littermates, respectively * p < 0.05: ** p < 0.01; *** p < 0.001 significantly different compared to 2-months-old mice from the same genotype. Color of the asterisk refers to the layer indicated in the figure legend. n = 5–6 per age group and genotype. Symbols indicate mean values and error bars standard error of means.

The size of GFAP-positive areas showed an uneven spatial distribution in the hippocampus that followed the distribution pattern of S100β-positive astrocytic somata (main effect: layer—CA1 region: F _3,87_ = 224.5, p *<* 0.001; CA3 region: F _4,112_ = 52.33, p *<* 0.001; Fig 4A and 4B). Unlike the number of astrocytic somata, the density of GFAP-positive areas significantly increased in older animals (age x layer interaction: CA1 region: F _9,87_ = 6.34, p < 0.001; CA3 region: F _12,112_ = 2.06, p < 0.05). We did not find a significant interaction between age, genotype and layer in the CA1 (F _9,87_ = 1.127, p > 0.05) or in the CA3 regions (F _12,112_ = 0.745, p > 0.05), which indicates that the age-related change in the distribution of GFAP-positive astrocytes within the hippocampal layers was not influenced by the genotype (Fig 4C and 4D).

### GABA-Cnr1^-/-^ mice show an increased number of GFAP-positive astrocytic processes

**Fig 5:**
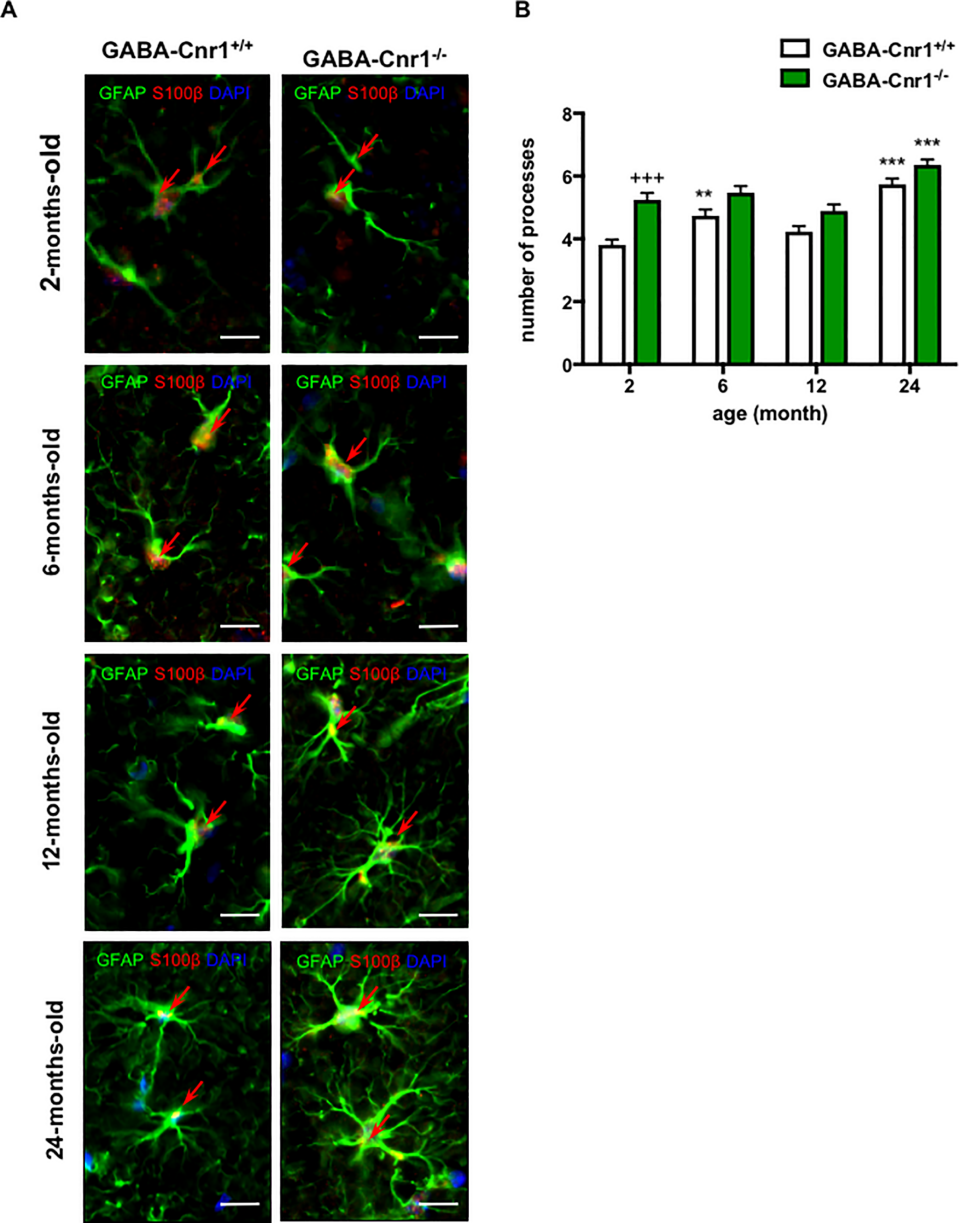
**(A)** Representative photomicrographs of GFAP- and S100β-immunopositive astrocytes in the stratum lacunosum moleculare layer of the CA1 hippocampal region of 2-, 6-, 12- and 24-months-old GABA-Cnr1^+/+^ and GABA-Cnr1^−/−^ mice, respectively. Scale bars, 10 μm. Red arrows indicate GFAP- and S100β-immunopositive co-staining. **(B)** The number of GFAP-positive processes visible 15 μm from the cell soma of the hippocampus in 2-, 6-, 12- and 24-months-old GABA-Cnr1^-/-^ and GABA-Cnr1^+/+^ littermates; n = 39–57 astrocytes per age and genotype. +++ p < 0.001 significantly different compared to GABA-Cnr1^+/+^ mice from the same age group. ** p < 0.01; *** p < 0.001 significantly differs from 2-month-old mice with the same genotype. Columns represent mean values, error bars standard error of means (SEM).

An increase in the size of GFAP-positive areas could be the result of enhanced astrocyte numbers or an increase in the number of GFAP-positive processes. To test the later possibility, we determined the number of GFAP-positive processes within 15 μm from the cell soma. As shown in Fig 5A and 5B, we found an age-related increase in the number of primary GFAP-positive processes (age effect: F _3,380_ = 25. 23, p < 0.001) and a significant genotype effect (genotype effect: F _1,380_ = 36.77, p < 0.001), with higher number of GFAP-positive processes in GABA-Cnr1^-/-^ mice. The genotype-age interaction failed to reach the level of significance (F _3,380_ = 2.090, p *=* 0.1011). Post hoc analysis of the data using the Bonferroni test revealed that the number of GFAP-positive processes in 2-month-old GABA-Cnr1^-/-^ mice was higher than in their wild-type littermates (Fig 5B).

### Age-dependent increase in astrocytic TNFα levels is more pronounced in GABA-Cnr1^-/-^ mice

**Fig 6:**
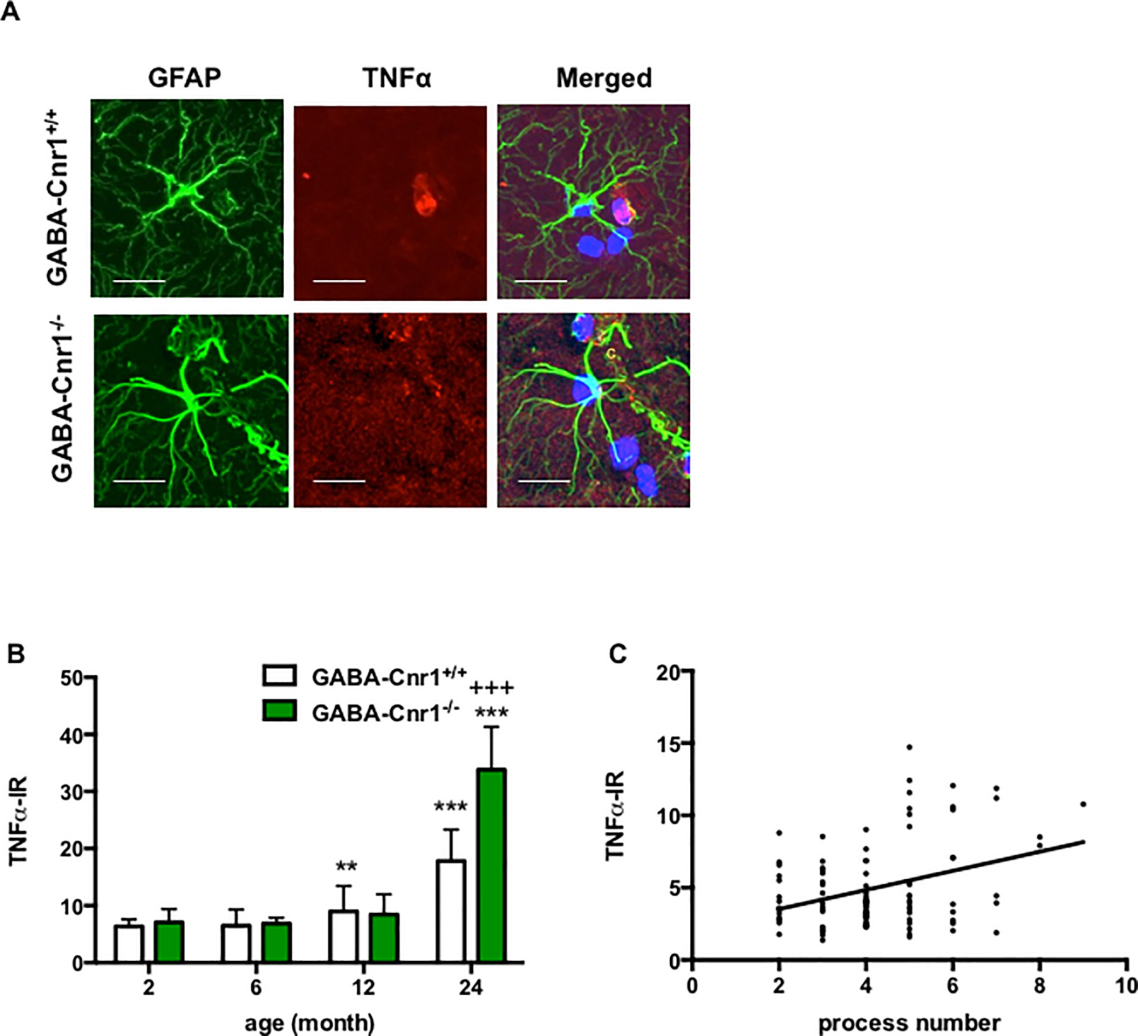
**(A)** Representative photomicrographs of GFAP- and TNFα-immunopositive astrocytes in the stratum lacunosum moleculare layer of the CA1 hippocampal region of 24-months-old GABA-Cnr1^+/+^ and GABA-Cnr1^−/−^ mice, respectively. Scale bars, 25 μm. Negative controls were stained without the anti-TNFα antibody. **(B)** Age-related increase in astrocytic TNFα levels is exacerbated in GABA-Cnr1^−/−^ mice. n = 42–60 per age and genotype. +++ p < 0.001 significantly different compared to GABA-Cnr1^+/+^ mice from the same age group. ** p < 0.01; *** p < 0.001 significantly differs from 2-months-old mice with the same genotype. Columns represent mean values, error bars standard error of means (SEM). **(C)** Positive relation between the number of GFAP-positive processes and TNFα-immunoreactivity in hippocampal astrocytes of 24 months-old GABA-Cnr1^-/-^ and GABA-Cnr1^+/+^ mice; n = 105.

To determine if CB1 receptor signaling on GABAergic neurons influences the age-related increase in the production of pro-inflammatory cytokines in astrocytes we compared the intensity TNFα immunoreactivity in GFAP-positive astrocytes of GABA-Cnr1^-/-^ and wild-type mice (Fig 6A). The amount of TNFα increased in ageing (age effect: F_3, 727_ = 103.3; p < 0.001) (Fig 6B). Importantly, this age-dependent increase was exacerbated in GABA-Cnr1^-/-^ mice (age x genotype interaction: F_3, 727_ = 13.67; p < 0.001) (Fig 6B). Finally, we determined the number of GFAP-positive process and TNFα levels in 105 randomly selected astrocytes in the hippocampus of 24-months-old wild-type and knockout mice. There was a significant correlation between number of GFAP-positive process and TNFα level (r = 0.303; p < 0.01), because astrocytes with higher number of GFAP-positive processes expressed also more TNFα (Fig 6C).

### Real-time PCR analysis of the hippocampal TNFα expression

**Fig 7:**
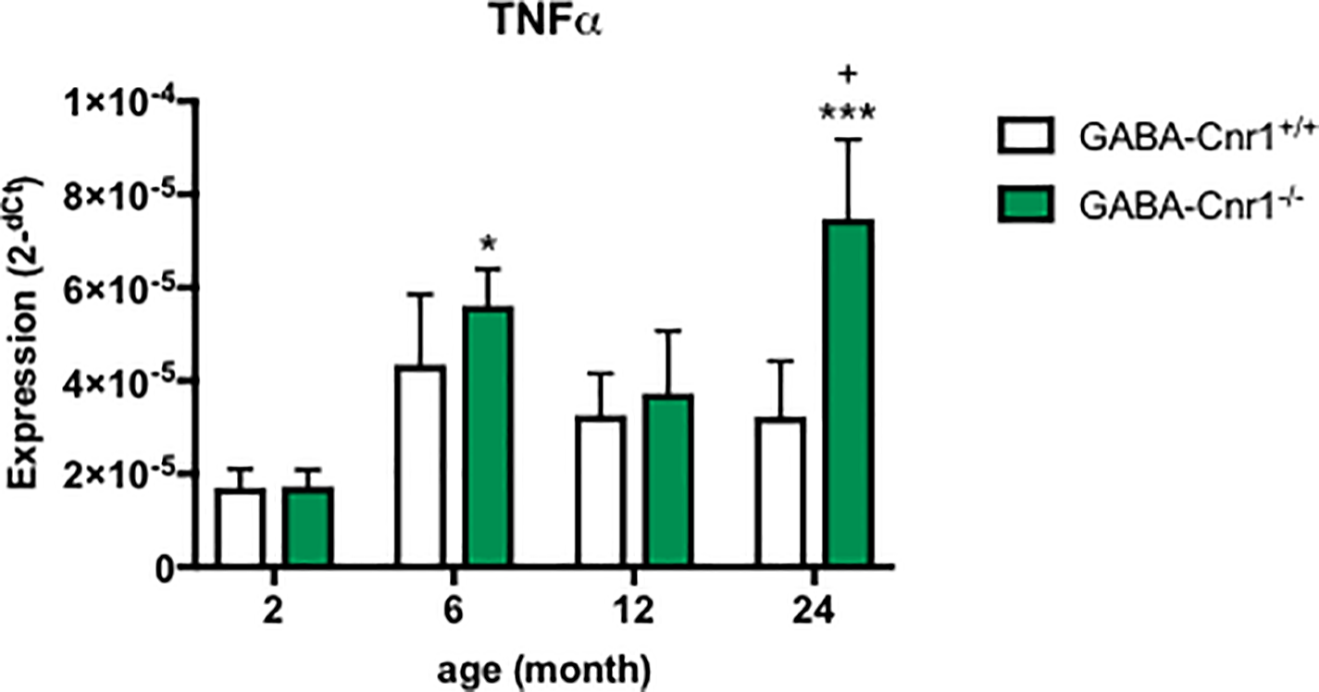
Hippocampal expression of TNFα. The age-related increase in expression is exacerbated in GABA-Cnr1^+/+^ mice. + p < 0.05 significantly different compared to GABA-Cnr1^+/+^ mice from the same age group. * p < 0.05; *** p < 0.001 significantly differs from 2-month-old mice with the same genotype. (n = 10–18 per age and genotype). Columns represent mean values, error bars standard error of means (SEM).

Lastly, we asked whether deletion of CB1 receptors from GABAergic neurons leads to an upregulation of TNFα expression in ageing in the hippocampus generally or specifically in the astrocytes. Age (F_3, 89_ = 5.523; p < 0.01) and also genotype (F_1, 89_ = 4.186; p < 0.05) influenced hippocampal TNFα expression without interaction between them (F_3, 89_ = 1.662; p > 0.05). As shown on Fig 7 the age-dependent increase failed to reach the level of significance according to Bonferroni-test in GABA-Cnr1^+/+^ mice, whereas the difference between 2-months-old and 6- as well 24-months-old GABA-Cnr1^-/-^ mice was significant. TNFα expression was significantly higher in 24-months-old GABA-Cnr1^-/-^ mice than in age-matched wild-types (Fig 7).

## Discussion

Ageing is associated with morphological and functional changes in astrocytes. We demonstrated here that GABAergic neurons significantly affect astrocyte changes in the ageing brain and that CB1 signaling on GABAergic neurons is necessary for the proper regulation of astrocyte activity.

An increase in GFAP-positive areas in the hippocampus has been observed during neuroinflammation and was found to be associated with an enhanced production of pro-inflammatory cytokines [48]. Our results confirm that ageing also leads to an increase in GFAP-covered areas [49,50] and demonstrate that it is enhanced by the deletion of CB1 receptors from GABAergic neurons. The age-related increase in the ratio of GFAP-positive areas was layer specific and most prominent in the hippocampal pyramidal cell layer, which has the lowest density of GFAP positive cells. As a consequence, the polarized distribution of GFAP staining was diminished in ageing, whereas the number and distribution of astrocytic somata remained constant. This indicates that astrocytes remained within their territories, but the area covered by their main cellular GFAP-positive processes was enlarged in a layer specific manner. Interestingly, our post hoc analysis showed that the increase in GFAP-positive areas was less pronounced the GABA-Cnr1^-/-^ mice, where the animals generally had a higher level of GFAP. In the hippocampus, the stratum lacunosum moleculare is the most GFAP-rich layer, and this was the only layer within the CA3 region where the age-related increase in GFAP-positive areas failed to reach the level of significance in GABA-Cnr1^-/-^ mice. We hypothesize that a ceiling effect is the reason of the reduced increase in hippocampal GFAP-positive areas in the GABA-Cnr1^-/-^ line generally and specifically in the stratum lacunosum moleculare layer of the CA3 region.

The increased arborization of GFAP-positive processes was more pronounced in GABA-Cnr1^-/-^ mice. Disrupted CB1 signaling on GABAergic neurons thus enhanced morphological changes in astrocytes typically associated with ageing. It remains to be determined if the number of astrocytic processes was increased or if the increased amount GFAP fills more processes and thus more processes become visible. Importantly, the increased number of GFAP-positive processes was associated with higher astrocytic TNFα levels suggesting the morphological changes are linked to a transition towards a pro-inflammatory activity. Indeed, we found a significant difference in the intensity of TNFα immunoreactivity in the astrocytes of wild-type and conditional knockout mice in the 24-months-old age group. Thus, the increasingly pro-inflammatory activity of aged astrocytes was exacerbated in GABA-Cnr1^-/-^ mice. Together these data show that cannabinoid signaling on GABAergic neurons influence age-related changes in astrocyte morphology, distribution and activity.

The hippocampus plays a crucial role in memory formation and shows numerous functional, structural, and morphological changes during normal ageing as well as in neurodegenerative disorders [51]. It has frequently been observed that the number of activated glial cells (astrocyte and microglia) increases in the hippocampus during ageing [22,52]. This has been linked to increased levels of inflammatory and oxidative stress molecules, which can lead to neuronal damage [14]. Neuronal damage/death can further enhance glial activation, thereby maintaining neuroinflammation in the brain [53–55].

An increased activity of astrocytes could be neuroprotective [56], but it can contribute to neuronal damage [57] depending on the context of pathology. Thus, it was shown that astrocyte activation was detrimental in a mouse model of taupathy [58], whereas they contributed to ischaemic tolerance in brain ischaemia [59] and remodeling by phagocytosis [60]. Besides removing cellular debris, astrocytes release functional mitochondria, which enter neurons and restore the failing energy support and strengthen intracellular survival pathways in the ischaemic brain [61]. It is not entirely known which pathways are responsible for the switch between neuroprotective and neurotoxic activity, but it was suggested that CD38 and cyclic ADP ribose signaling contributes to mitochondria transfer between astrocytes and neurons [61], whereas neuronal release of ephrin-B1 induces an anti-inflammatory expression profile partly through STAT3 pathway [62]. Interestingly, direct injection of interleukin-1β led to an upregulation of both neuroinflammatory chemokines and neuroprotective growth factors [63]. Whether activated astrocytes in the ageing brain are neurotoxic or neuroprotective is not known. Nevertheless, one of the most characteristic change in ageing astrocytes is the up-regulation of GFAP, which could be a sign of neurotoxic activity: Mice with a genetic deletion of GFAP showed a reduced sensitivity to cytotoxic insults [64], whereas the genetic ablation of reactive astrocytes led to an enhanced neuronal degeneration [65]. A recently published finding, that the expression profile of astrocytes in the ageing brain is similar as the profile of astrocytes in the brain of mice injected with the highly inflammatory bacterial lipopolysaccharide (LPS) suggests that astrocytes in the ageing rain can contribute to neuronal deficits [15]. Previous studies suggest that astrocytes modulate synaptic [66] and network activities [67,68]. Thus, an increase in astrocyte territorial volume in old mice may directly influence hippocampal activity. Both astrocytes and microglia cells express enzymes involved in the synthesis and degradation of endocannabinoids [26,69,70], and produce cannabinoids such as anandamide [71], 2-AG [26,72] and palmitoylethanolamide [73]. Thus, endocannabinoids with glial origin can bind and activate neuronal cannabinoid receptors and thus contribute to the neuron-glia communication [74,75]. Although astrocytes in the adult mouse brain express cannabinoid receptors only at low levels [74,76,77], CB1 receptors were found to modulate important astrocyte functions [78–80]. Microglia also express cannabinoid receptors at low levels in non-activated scavenging microglia, but CB2 expression is significantly increased by pro-inflammatory stimuli [74].

Deletion of CB1 receptors led to an upregulation of microglia activity, indicating that cannabinoid signaling is important to maintain an anti-inflammatory milieu in the brain [22]. In the hippocampus of 12-month-old Cnr1^-/-^ mice the total number of microglia, the ratio of activated microglia and the expression of the pro-inflammatory cytokine interleukin 6 was significantly higher. Pharmacological studies also showed that increased activity of the cannabinoid system is generally anti-inflammatory. Elevation of anandamide levels [81,82], or activation of the cannabinoid receptor by synthetic receptor agonists [83,84] inhibits the production of pro-inflammatory mediators and reduces microglial migration in vitro. This effect may contribute to the beneficial effect of the cannabinoid system against neurodegeneration [85].

Importantly, cannabinoids can exert their anti-inflammatory effect directly by binding glial cannabinoid receptors [86–88] and also indirectly, by modulating neuronal activities [22]. Our results now show that lack of CB1 receptor on GABAergic neurons disturbed neuron-glia communication and led to an enhanced pro-inflammatory activity of the astrocytes. GABAergic neurons can directly render astrocytes to a more activated state through direct interactions. However, astrocytes react to an enhanced microglial activity by changing their phenotype and by becoming neurotoxic through the production of pro-inflammatory cytokines and reducing neuronal support [89]. It is possible that the observed changes in astrocyte morphology and activity in GABA/Cnr1^-/-^ mice are an indirect consequence of increased pro-inflammatory microglial activity [22]. We cannot exclude the possibility that deletion of CB1 receptors from the GABAergic neurons altered the level of astrocytic CB1 receptors. However, in GABA/Cnr1^-/-^ mice the expression of CB1 receptors in glutamatergic neurons [29] and the number or distribution of GABAergic neurons [31] is not affected.

During normal, healthy ageing the level of the major endogenous cannabinoid 2-arachidonoylglycerol [90,91] and the coupling of the CB1 receptors to G_i_ protein [90,91] are significantly reduced in old individuals. Our results now suggest that the age-related decline in cannabinoid system activity could be responsible or at least contribute to the development of astrocyte ageing [92].

Multiple lines of evidence suggest an important role of cannabinoid signaling in ageing [92,93]. Impaired intercellular signaling is one of the key hallmarks of ageing [94], therefore one of the mechanisms by which cannabinoids may exert their anti-ageing effect is the maintenance and support of neuron-neuron [27] and neuron-glia [87,95,96] communication. Our results support this hypothesis, showing that disturbed cannabinoid signaling on GABAergic neurons affects astrocytes in an age-dependent manner that renders them more activated.
